# Splicing Modulation as a Promising Therapeutic Strategy for Lysosomal Storage Disorders: The Mucopolysaccharidoses Example

**DOI:** 10.3390/life12050608

**Published:** 2022-04-19

**Authors:** Juliana Inês Santos, Mariana Gonçalves, Liliana Matos, Luciana Moreira, Sofia Carvalho, Maria João Prata, Maria Francisca Coutinho, Sandra Alves

**Affiliations:** 1 Research and Development Unit, Department of Human Genetics, National Institute of Health Doutor Ricardo Jorge (INSA I.P.), Rua Alexandre Herculano, 321, 4000-055 Porto, Portugal; mariana.goncalves@insa.min-saude.pt (M.G.); liliana.matos@insa.min-saude.pt (L.M.); luciana.moreira@insa.min-saude.pt (L.M.); sofia.carvalho@insa.min-saude.pt (S.C.); francisca.coutinho@insa.min-saude.pt (M.F.C.); sandra.alves@insa.min-saude.pt (S.A.); 2 Biology Department, Faculty of Sciences, University of Porto, Rua do Campo Alegre, 4169-007 Porto, Portugal; mprata@ipatimup.pt; 3 Center for the Study of Animal Science, CECA-ICETA, University of Porto, Praça Gomes Teixeira, Apartado 55142, 4051-401 Porto, Portugal; 4 Centre for the Research and Technology of Agro-Environmental and Biological Sciences (CITAB), Inov4Agro, University of Trás-os-Montes and Alto Douro, 5000-801 Vila Real, Portugal; 5 Faculty of Farmacy, University of Coimbra, 3000-548 Coimbra, Portugal; 6 i3S-Institute of Research and Innovation in Health/IPATIMUP-Institute of Molecular Pathology and Immunology of the University of Porto, Rua Alfredo Allen, 208, 4200-135 Porto, Portugal

**Keywords:** lysosomal storage diseases (LSDs), mucopolysaccharidoses (MPSs), RNA-based therapies, antisense oligonucleotides (ASOs), splice-switching oligonucleotides (SSOs), U1 snRNA (small nuclear RNA)

## Abstract

Over recent decades, the many functions of RNA have become more evident. This molecule has been recognized not only as a carrier of genetic information, but also as a specific and essential regulator of gene expression. Different RNA species have been identified and novel and exciting roles have been unveiled. Quite remarkably, this explosion of novel RNA classes has increased the possibility for new therapeutic strategies that tap into RNA biology. Most of these drugs use nucleic acid analogues and take advantage of complementary base pairing to either mimic or antagonize the function of RNAs. Among the most successful RNA-based drugs are those that act at the pre-mRNA level to modulate or correct aberrant splicing patterns, which are caused by specific pathogenic variants. This approach is particularly tempting for monogenic disorders with associated splicing defects, especially when they are highly frequent among affected patients worldwide or within a specific population. With more than 600 mutations that cause disease affecting the pre-mRNA splicing process, we consider lysosomal storage diseases (LSDs) to be perfect candidates for this type of approach. Here, we introduce the overall rationale and general mechanisms of splicing modulation approaches and highlight the currently marketed formulations, which have been developed for non-lysosomal genetic disorders. We also extensively reviewed the existing preclinical studies on the potential of this sort of therapeutic strategy to recover aberrant splicing and increase enzyme activity in our diseases of interest: the LSDs. Special attention was paid to a particular subgroup of LSDs: the mucopolysaccharidoses (MPSs). By doing this, we hoped to unveil the unique therapeutic potential of the use of this sort of approach for LSDs as a whole.

## 1. Introduction

**Figure 1:**
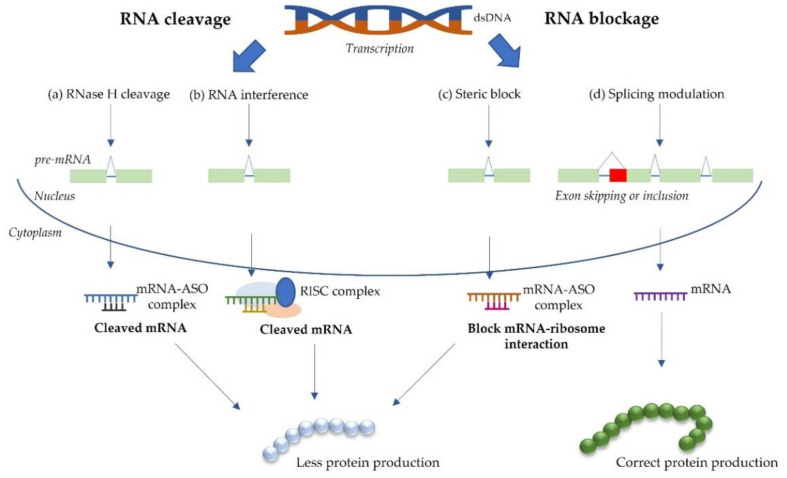
Schematic representation of the different mechanisms of action of antisense oligonucleotides (ASOs). ASOs can impact gene expression in different ways, either through RNA cleavage (**a**,**b**) or RNA blockage (**c**,**d**). RNA cleavage (or degradation) approaches include (**a**) RNAse H-mediated mRNA degradation and (**b**) RNA interference (RNAi), while RNA blockage approaches may promote (**c**) sterick block of ribosome binding and (**d**) splicing modulation. The green rectangles represent the coding exonic regions and the blue lines represent the non-coding intronic regions from the pre-mRNA. The red square represents the mutated region of the exon. The dashed lines that form a triangle represent the normal splicing pattern of the pre-mRNA. Abbreviations: ASO, antisense oligonucleotide; mRNA, messenger RNA; pre-mRNA, pre-messenger RNA; RISC, RNA-inducing silencing complex (Adapted from [2]).

The somehow recent revolution in RNA biology has led to the recognition of the multiple roles that this molecule may assume within a cell through the identification of new RNA classes that have previously unanticipated functions. This better understanding of basic RNA biology has been accompanied by a parallel revolution in the use of RNA-based strategies for therapeutic purposes [1]. All of a sudden, RNA-based drugs opened a whole new perspective on therapeutic approaches for previously untreatable diseases by entering the pharmacopoeia and greatly expanding the universe of druggable targets (Figure 1).

Among this promising class of drugs, those that target the splicing process are probably the most widely studied and for which there are five approved drugs for two different diseases [3]. Splicing defects are particularly tempting as therapeutic targets because mutations in the consensus sequences at the borders of introns and exons are a common cause of human genetic diseases. Furthermore, those defects tend to result in the complete loss of function of the protein in question, thus underlying severe pathology [4].

Splicing defects in different genes have been identified as one of the underlying genetic causes of a huge number of genetic diseases of different etiologies. Among those disorders are countless rare diseases of monogenic origin, including the lysosomal storage diseases (LSDs) that were our major focus of interest. LSDs are a particular subset of genetic diseases that can benefit greatly from even the slightest increase in protein function [5]. The vast majority of LSDs are autosomal recessive, even though three X-linked diseases are also known. Still, few disease-specific therapies exist for this vast and heterogeneous group of disorders and even when they do exist, it is now well-recognized that there are some major drawbacks to the existing approaches, such as their inability to act on neurological symptoms [6]. Unfortunately, a great majority of LSDs have a significant neurological component, which is the dominating clinical effect of the disease in a number of disorders, although it is merely one element of a more generalized pathology in others [7]. Among the LSDs that are still lacking effective treatment, a major group is the mucopolysaccharidoses (MPSs). The MPSs comprise a group of 11 disorders and each one is caused by defects in any of the enzymes that are involved in the stepwise degradation of glycosaminoglycans (GAGs), which lead to the progressive storage of those compounds. This storage, along with other pathogenic mechanisms, triggers several clinical consequences of wide phenotypic variability [8]. Interestingly, even patients that suffer from the same disease can present with extremely different phenotypes that are associated with enzyme activity levels: some patients, who have null or residual enzyme activity, present with early onset severe phenotypes; others, who retain significantly higher residual enzymatic activity, show a much more slowly progressing disorder with a later onset. This means that even a slight recovery in enzyme activity (which can be promoted by the recovery of the normal splicing) can be enough to have a clinical impact [9,10]. Of all MPS-causing mutations, a large percentage affect the pre-mRNA splicing process. Altogether, this makes MPSs excellent candidates for splicing correction therapeutics. Nevertheless, despite the immense potential that these approaches hold for this group of diseases, there are only a few works so far that have attempted splicing modulation approaches for these disorders.

In this work, we address this issue and comment not only on the potential of these drugs but also on the hurdles they must overcome. We start by explaining how splicing can be experimentally modulated for therapeutic purposes. In order to do so properly, we begin by briefly summarizing the normal splicing process and the possible consequences of its disruption. Then, we introduce the currently approved therapeutic approaches that modulate splicing and their mechanisms of action, even though they were not designed for LSDs. Finally, we bring the focus onto our diseases of interest: the MPSs. After an overview of their major clinical features and molecular bases, we outline the contribution of splicing defects to each of the individual diseases. Then, we discuss how some of them have been approached for therapeutic purposes and summarize the published preclinical studies that have assessed the feasibility of recovering pre-mRNA splicing mutations as a way to recover defective enzyme activity. Finally, we comment on the future of splicing therapeutics and the major issues that may hamper their transfer to the clinics and highlight a few strategies that could be used to overcome those hurdles.

## 2. Splicing: How It Works and How It Can Be Modulated

### 2.1. The Splicing Process: Machinery and Mechanisms

It is well known that eukaryotic gene(s) expression requires a series of highly regulated sequential steps in which non-coding introns are removed from the precursor messenger RNA (mRNA) molecule while the exons, or coding sequences, are joined together, which results in mRNA maturation being translated into protein. This well-known process is called splicing and is carried out by the spliceosome.

RNA splicing was initially discovered in the 1970s and it overturned years of research in the field of gene expression [11,12]. Its major effector, the spliceosome, functions in a complex and dynamic assembly–disassembly cycle in which five small nuclear ribonucleoprotein (snRNP) complexes (U1, U2, U4/U6 and U5) recognize and assemble on each intron to ultimately form a catalytically active spliceosome. An early event in the exon definition is the recognition of the 5′ donor splice site (ss) by the U1 snRNP, which is followed by the binding of splicing factor 1 (SF1) to the branch point and the binding of the U2 auxiliary factor heterodimer (U2AF 65/35) to the polypyrimidine tract (Py) and 3′ss, originating the E complex [13,14]. After that, SF1 is replaced by the U2 snRNP at the branch point, originating the A complex, which allows for the interaction between U1 snRNP and U2 snRNP across the exon [13,15]. Then, the U4, U5 and U6 snRNPs are recruited as a preassembled complex, which leads to the formation of the B complex. Afterward, the interaction between U4 and U6 is disrupted and the U6 snRNP base pairs with the 5′ss, thereby displacing U1 snRNP from its initial location and releasing it from the complex along with the U4 snRNP [16]. At the same time, U6 snRNP interacts extensively with U2 snRNP, which brings the 5′ss and the branch point into close proximity. This allows for the first step of splicing to take place, which originates the C complex, which contains the free upstream exon and the intron–exon lariat intermediate [15]. This complex completes the second step of the splicing reaction and releases the intron and joins the exons together to form the mature mRNA, while the U2, U5 and U6 snRNPs are also released from the complex and recycled for future splicing reactions [15,17,18].

Although the spliceosome drives pre-mRNA processing with great complexity and fidelity, this is quite a flexible mechanism under the strong regulation by both *cis*- and *trans*-acting elements. The role of *cis*-acting regulatory sequences and RNA-binding protein splicing factors, which recognize and bind to those sites, compose a common mechanism for setting up and maintaining alternative splicing (AS) patterns. Heterogeneous nuclear ribonucleoproteins (hnRNPs) and serine and arginine-rich (SR/AR) proteins in the spliceosome regulate either splicing repression by binding intronic splicing silencers (ISS) and exonic splicing silencers (ESS) or splicing activation by binding intronic splicing enhancers (ISE) and exonic splicing enhancers (ESE) [14,15,19].

**Figure 2:**
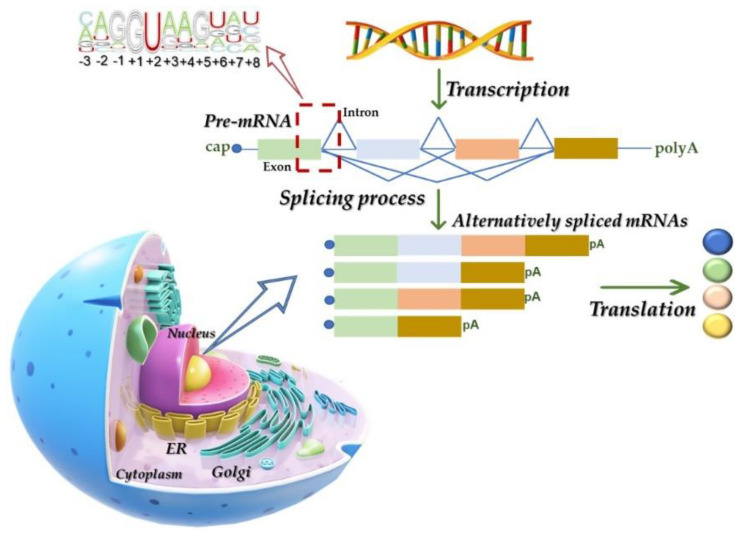
Simplified overview of the splicing process. The alternative splicing (AS) process generates mature mRNAs with different exon combinations, which results in the production of different protein isoforms from the same mRNA. Abbreviations: ER, endoplasmatic reticulum; mRNA, messenger RNA; pre-mRNA, precursor mRNA.

AS is a process through which a single precursor mRNA can generate a number of alternative mRNAs, thereby allowing for considerable proteomic diversity and complexity [20,21]. It is currently estimated that nearly 95% of human multi-exonic genes are alternatively spliced, thus giving rise to different protein isoforms. AS mechanisms include: exon skipping, intron retention, mutually exclusive exons and alternative donor 5′ss and acceptor 3′ss [19]. Furthermore, alternative polyadenylation sites and the alternation of the initial exons due to alternative promoter usage can also contribute to AS. In addition, AS can be regulated at the transcription level and in the chromatin structure (Figure 2).

A detailed description of the AS process and regulation, which was beyond the scope of this review, can be found in a series of papers that have been published elsewhere [15,22,23,24]. A variety of therapeutic strategies, such as small molecules and antisense oligonucleotides (ASOs) as well as genome editing through the use of CRISPR/Cas9, have promising future interventions for the amelioration of the disease-causing effects of human mutations on the patterns of AS. Over the following sections, we briefly describe some of the interventions with a special focus on those that are currently approved for commercial use.

### 2.2. RNA-Based Approaches for Splice Modulation

In general, antisense-mediated splicing modulation is a tool that can be exploited in several ways to provide a potential therapy for rare genetic diseases [25]. It is an extremely versatile approach because it can not only promote the correction of cryptic splicing and the modulation of AS, but also the restoration of the open reading frame. Ultimately, it can even induce protein knockdown. This means that splicing modulation approaches can actually go far beyond the correction of individual splicing mutations (such as those that we focus on subsequent sections: see Section 4). Additionally, it may also rely on different effectors, or tools, from antisense oligonucleotides (ASOs) for splicing-switching to synthetic U1 snRNAs (small nuclear RNAs). The most widely known tools that are used to promote splicing correction/modulation are ASOs.

ASOs were first reported by Stephenson and Zamecnik in 1978 [26]. ASOs are short synthetic oligonucleotides (15–30 nucleic acid length) designed complementary to sense strand of mRNA and efficient laboratory tools that can regulate the expression of specific genes through an efficient modulation of the splicing process [27]. When designed to target the splice site or its auxiliary sequences, which leads to mRNA repair and the restoration of protein function and modifies the outcome of the splicing reaction, they are called splice-switching ASOs or splice-switching oligonucleotides (SSOs). These ASOs are able to sterically block relevant motifs in the pre-mRNA without promoting its degradation.

Numerous studies have investigated the therapeutic potential of ASOs in *in vitro* cell models, animal disease models and human clinical trials. Even though a complete overview of all of these studies clearly fell outside of the scope of this review, we briefly discuss the approved therapeutic strategies to treat diseases using ASOs. By doing so, we hope to unveil the full potential of this somewhat novel class of drug and show how life-changing these molecules can be for patients who harbor different genetic mutations, provided that a number of requirements are met.

The demonstration that an ASO drug can successfully promote the correction of its targets *in vivo* paved the way for the clinical trials of ASOs as a treatment for a variety of diseases, especially rare diseases such as Duchenne muscular dystrophy (DMD) and spinal muscular atrophy (SMA). Currently, there are a number of approved drugs for these pathologies, all of which are capable of manipulating the pre-mRNA splicing process: Eteplirsen (EXONDYS 51™, Sarepta Therapeutics, Cambridge, MA, USA) [28,29]; Golodirsen (Vyondys 53™, Sarepta Therapeutics, Cambridge, MA, USA) [30]; Viltolarsen (Viltepso^®^, NS Pharma, Paramus, NJ, USA) [31]; and Casimersen (Amondys 45™, Sarepta Therapeutics, Cambridge, MA, USA) [32] for the DMD and Nusinersen (Spinraza^®^, Biogen, Cambridge, MA, USA) [33,34] for SMA (Table 1).

**Figure 3:**
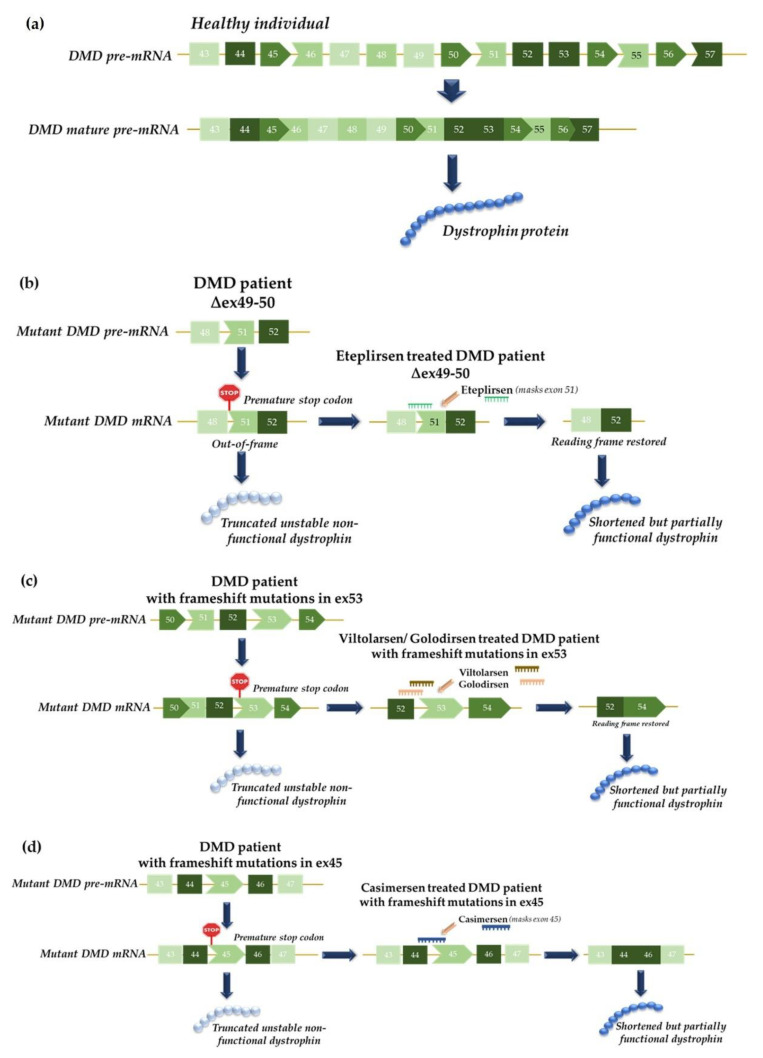
Mechanism of action of exon skipping therapy for Duchenne muscular dystrophy (DMD): (**a**) schematic representation of the normal splicing of the *DMD* gene in healthy individuals who produce normal dystrophin protein. In general, treatment of DMD with antisense oligonucleotides (ASOs) promotes selective exon skipping in order to restore the reading frame and produce a truncated but partly functional dystrophin protein. Different drugs are available for the different mutations that affect a number of *DMD* exons: (**b**) Eteplirsen, for DMD patients with deletions spanning exons 49 and 50; (**c**) Viltolarsen/Golodirsen, for DMD patients with frameshift mutations in exon 53; and (**d**) Casimersen, for DMD patients with frameshift mutations in exon 45. Abbreviations: ∆49-50, deletion of exons 49 and 50; DMD, Duchenne muscular dystrophy; mRNA, messenger RNA; pre-mRNA, precursor mRNA.

DMD is an X-linked genetic disease that is characterized by the absence of the dystrophin protein in muscle fibers, which is manifested by progressive muscle degeneration and weakness. Approximately two thirds of DMD cases present deletion mutations in the *DMD* gene, which is composed of 79 exons (the largest known human gene) [35]. Becker muscular dystrophy (BMD) is a mild disease that is caused by dystrophin truncations and not by its absence. To produce mild phenotypes, such as BMD, a strategy that can generate a truncated but functional dystrophin protein would be a reliable tool. Thus, the skipping of exons to correct DMD-linked mutations can reduce the severity of the disease and produce a phenotype that is similar to that of BMD [36]. Eteplirsen, which is a 30-nucleotide phosphorodiamidate morpholino oligomer (PMO), binds to the 5′ss of exon 51, which leads to it being skipped (Figure 3b). Thus, an in-frame transcript is produced that allows for the formation of an internally truncated but functional dystrophin protein [36,37]. Eteplirsen can only be used for patients who are amenable to exon 51 skipping, which accounts for 13% of the DMD patient population [38]. In September 2016, this drug received approval from the US Food and Drug Administration (FDA), which made it the first ASO to be approved for DMD and the first approved exon skipping ASO to be used for humans [38].

More recently, in 2019, another ASO drug was approved to treat this disease: Golodirsen. This is a 25-mer PMO that binds to the exon 53 of the *DMD* gene and causes it to be skipped, thereby avoiding the deleterious loss-of-function frameshifting mutations [30,39]. It was only approved for males with mutations that are amenable to exon 53 skipping. Then, in 2020, yet another drug for the treatment of DMD patients with the same characteristics was approved by the FDA: Viltolarsen, which is a 21-mer PMO that also binds to exon 53 and causes it to be skipped [31,40] (Figure 3c). In both cases, the skipping of this exon restores the reading frame and leads to the production of an internally truncated but partially functional dystrophin protein [41]. Both drugs are suitable for 8% of DMD patients. Finally, in 2021, an ASO from the PMO subclass was developed by Sarepta Therapeutics for the treatment of DMD in patients who have a mutation of the *DMD* gene that is amenable to exon 45 skipping: Casimersen. Casimersen was designed to bind to the exon 45 of the *DMD* gene pre-mRNA and leads to the production of an internally truncated but functional dystrophin protein [32] (Figure 3d).

Altogether, ASOs that address the primary genetic defect of DMD are among the first generation of therapies tailored to overcome specific genetic mutations in humans. They represent paradigm-forming approaches to medicine that may lead to life-changing treatments for those affected by this relentlessly progressive and fatal disease [42].

**Figure 4:**
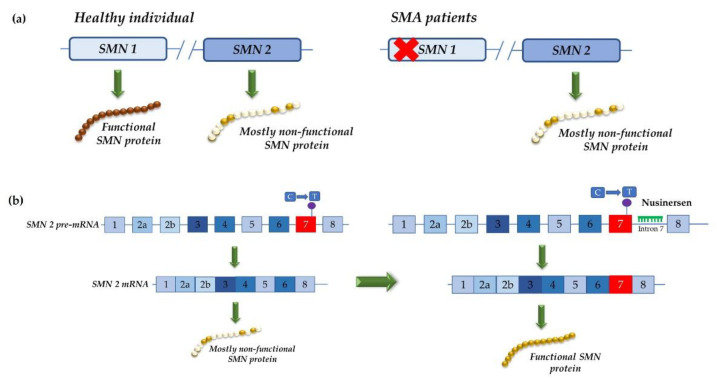
Mechanism of action of exon inclusion therapy for spinal muscular atrophy (SMA): (**a**) overview of the molecular basis of SMA. Humans have two *SMN* genes: *SMN1*, which gives rise to a functional SMN protein and *SMN2*, which has a C to T mutation in exon 7 that does not affect the protein sequence but does affect the pre-mRNA splicing, thereby giving rise to an unstable isoform that is rapidly degraded. In healthy individuals, the presence of a functional SMN protein that is encoded by the *SMN1* gene assures the assembly of the cellular machinery that is needed to process pre-mRNA. In SMA patients, mutations in the *SMN1* gene prevent the production of a functional SMN protein: (**b**) Nusinersen targets and blocks the intronic splicing silencer (ISS) in intron 7, which induces the inclusion of exon 7 in the *SMN2* mRNA. Abbreviations: C, cytosine; mRNA, messenger RNA; pre-mRNA, precursor mRNA; SMA, spinal muscular atrophy; SMN, survival motor neuron; T, thymine.

SMA is another disorder that has greatly benefited from the development of splice modulation therapeutics. SMA is an autosomal recessive neuromuscular disease that is caused by mutations and deletions in the survival motor neuron 1 (*SMN1*) gene, which results in the progressive loss of alpha motor neurons in the anterior horn of the spinal cord [43]. A second *SMN* gene exists in human genome: the *SMN2* that has a C to T mutation in exon 7. This single nucleotide change does not affect the protein sequence but it does affect the pre-mRNA splicing, which gives rise to an unstable isoform that is rapidly degraded [44,45] (Figure 4a).

Taking this into account, Cartegni and colleagues showed that a 2′-O-methoxyethyl (2′MOE) phosphorothioate-modified ASO can efficiently correct *SMN2* exon 7 splicing both *in vitro* and *in vivo* [43,46,47]. By targeting and blocking the intron 7 ISS, Nusinersen induces the inclusion of exon 7 in the *SMN2* mRNA. This ASO was approved by the FDA in December 2016 [48] (Figure 4b). Together with the two other approved drugs for SMA replacement therapy, Nusinersen has provided a life-changing treatment option for SMA patients and their families. It extends life expectancy and allows patients to reach motor milestones that would previously have been unachievable [49].

The second major approach for the modulation of the splicing process, both *in vitro* and *in vivo*, is the use of synthetic U1 snRNAs designed to recognize mutant 5′ss, thus restoring complementarity. The first step of the splicing process requires the 5′ end of the U1 snRNA to interact by complementarity with the moderately conserved sequence of the 5′ss [20]. This implies that any mutation in this site may compromise the binding of the U1 snRNA and prevent spliceosome assembly, thus inhibiting the subsequent splicing process. Therefore, these sorts of variants usually cause disease.

Over the last decade, U1 snRNAs with a modified 5′ tail that base pair exactly to the mutant splice site have been used to correct 5′ss mutations, abolishing the skipping of some the exons that they originally caused. Another type of modified U1 snRNAs are the so-called exon-specific U1 snRNAs (ExSpe U1s), which have also been tested in different *in vitro* and *in vivo* approaches that have shown their therapeutic potential [20,50,51,52]. Recently, Balestra and colleagues published the *in vivo* proof of principle for the correction potential of compensatory U1 snRNAs in hereditary tyrosinemia type I [50]. Nevertheless, this approach is not yet available as a therapeutic option and more studies are needed before its translation into the clinic.

The combined use of ASOs and U1snRNAs is also under consideration. In fact, a combined treatment using ASOs and engineered U1 snRNAs has shown the highest therapeutic efficacy for correcting mutation-induced splicing defects in Bardet–Biedl syndrome [51]. This recent observation has shown that there may be an advantage in the use of these two therapeutic approaches with complementary effects for the improvement of treatment efficacies.

Among the monogenic diseases, which may benefit from either sort of splice-modulation therapeutics are the LSDs, a group of life-threatening disorders, which are further addressed in the following sections.

### 2.3. Hurdles

Despite its promise, the development of RNA therapeutics has faced several major hurdles over recent decades, namely: (1) the rapid degradation of exogenous RNAs by ubiquitous endogenous RNases; (2) the challenging delivery of negatively charged RNA molecules across hydrophobic membranes; and (3) the strong immunogenicity of synthetic RNAs, which ends up causing cell toxicity and impairing translation. These hurdles have been substantially overcome with recent advancements in RNA biology, bioinformatics, separation science and nanotechnology, all of which have greatly facilitated the recent rapid development of RNA therapeutics as a whole [53].

However, there are several challenges that may still hinder the prompt clinical translation of some RNA drugs. Most of these challenges are common to all types of RNA drugs, but others are specific to those that are aimed at splicing modulation.

For example, the development of proper models to assess the sequence-dependent efficacy and safety of ASOs is still a pending issue [54]. This is particularly relevant for the splicing modulation approaches designed to correct specific disease-causing mutations that affect the normal splicing process, the so-called splicing mutations. Ideally, the preclinical development of that sort of drugs would require the development of animal models that carry the specific splicing mutations. Importantly, however, an alternative exists for a few specific approaches that does not require these mutation-specific models. In fact, for the therapies that rely on the promotion of the skipping of a specific exon, it is possible to use wild animals instead of mutation-specific models.

Then, there is the question of the species-specific sequence differences between orthologous genes. SSOs and U1snRNA-based therapies are sequence-specific approaches that aim to interfere with the splicing mechanism and they are specifically designed to recognize a certain target sequence in the human genome. Unfortunately, most of our sequences of interest are not completely conserved among different species. Therefore, the molecules designed to target a human sequence cannot be directly assessed in an animal model [55]. This means that for *in vivo* assessments, it is not usually possible to use exactly the same SSOs or U1snRNA sequences that are used for human cells. It is always necessary to design species-specific SSOs and U1snRNAs (i.e., specifically designed for animal sequences, which are orthologous to the human genes under study). This is actually the standard approach for *in vivo* ASO studies and most of the currently approved ASOs relied on the *in vivo* assessment of animal responses to slightly modified molecules, which were designed to match the orthologue sequences. This sort of *in vivo* studies may provide relevant safety and toxicity data, but it relies on the premise that the consensus splice site sequences in mice and humans are highly conserved and comparable. Still, some small changes in these patterns have been described [56].

The alternative would be to generate humanized animal models, an approach that is both time- and resource-consuming and may contribute to a substantial increase in the drug development time while requiring additional funding. Furthermore, the generation of a humanized animal model for every mutation that needs to be targeted is neither feasible nor ethical and may not always recapitulate the human molecular and/or physiological phenotypes [54,55].

The last and probably the major challenge that could hinder the broader clinical translation of this category of drugs is their inefficient delivery to the target tissues. This is not only true for splicing modulation but also for every other RNA-based approach. In general, the delivery of ASOs and any other RNA-based drugs to target tissues is relatively poor after systemic delivery. Nevertheless, relevant increases in the efficiency of ASO delivery have been achieved over recent years through chemical modification and conjugation to other moieties, as well as the development of new chemical backbones. Furthermore, many teams have been working on the development of effective drug delivery systems, which ultimately enhance the delivery of drugs to the target sites of pharmacological action. Among these systems, lipid nanoparticles (LNPs) and/or adeno-associated viruses (AAVs) are probably the most well known (reviewed in [57]). Nevertheless, the latest advances in ASO technology have been coupled with the surprising finding that despite being highly charged and large, ASOs distribute widely throughout the CNS when they are delivered to the cerebral spinal fluid via intrathecal (IT) delivery, which is safe and well tolerated. This peculiarity (contrary to other RNA therapies, such as siRNAs and U1snRNAs vectors) has greatly enabled the application of ASOs as a therapeutic strategy for CNS disorders, many of which currently have no treatment [58]. Remarkably, IT ASO administration has already been implemented for the treatment of SMA and has produced safe and tolerable results [58,59]. An ASO that targets ALS and is delivered via IT was also recently administered to one patient [60].

Over the last decade, huge successes have also been documented for therapies that target hepatocytes and in which GalNAc conjugation and LNP technology allow for the targeted delivery of drugs with outstanding results, which has resulted in approval being granted for several clinical indications. These examples of how specific and well-designed drug delivery technologies can be used to overcome the targeting hurdles have provided a new impetus to the RNA-based therapeutics field, which will certainly contribute to fostering research and accelerating discoveries about extra-hepatic delivery (reviewed in [61]). Another drawback is the high exposure of certain organs upon the systemic delivery of AONs. For instance, after the intravenous injection of AONs, a significant proportion is absorbed by the liver and kidneys. This limits their biodistribution to other tissues and results in a toxic effect within these organs. Importantly, however, many of the liver and kidney injuries were found when using high and not clinically relevant doses of AONs. Obviously, the design and manufacture of efficient delivery systems is not the only hurdle. Their safety, both alone and in combination with RNA-based drugs, is also paramount [61].

## 3. Treatment Strategies for LSD Patients: MPSs in the Spotlight

### 3.1. Lysosomal Storage Diseases

Lysosomal storage diseases (LSDs) are a group of about 70 monogenic and hereditary diseases of lysosomal catabolism. The majority of them are inherited in an autosomal recessive manner, but three diseases are X-linked. These disorders have a combined incidence of around 1:7700 but, according to several authors, this figure may be as high as 1:3000 or even 1:1500 when all LSDs are considered [62,63]. LSDs occur when a mutation, or more than one mutation, occurs in genes that code for proteins that are important for lysosomal function (i.e., lysosomal proteins, in the majority cases), thus affecting their function. This results in lysosomal malfunction and the gradual storage of the undegraded/partially degraded substrates inside the lysosome, which ultimately results in cell dysfunction and death [64,65].

Frequently, LSDs present as pediatric neurodegenerative diseases [66]. However, as they are heterogeneous disorders, depending on the gene defect and on the biochemical nature of the stored substrates, lysosomal storage defects can cause skeletal dysmorphia, due to bone pathology, and central nervous sys-tem (CNS) defects, in addition to symptoms affecting many other organs..

LSD diagnosis is usually based on the clinical symptoms of patients, followed by the confirmation of increased storage and genetic alterations through several diagnostic tests, such as enzymatic analysis and gene sequencing. More recently, diagnosis through next generation sequencing (NGS) has become routine, which greatly reduces the time from the initial presentation of symptoms to the diagnosis of the disease [67,68].

Based on the type of disorder and the age of diagnosis, LSDs can be classified into congenital or infantile, late-infantile, juvenile and adult types. Usually, the earlier the symptoms appear, the more severe the disease presentation.

Treatment strategies for LSDs include: enzyme replacement therapy (ERT), which consists of providing the missing/defective enzyme; substrate reducing therapy (SRT), in which the synthesis of the accumulated substrates is reduced; hematopoietic stem cell transplantation (HSCT), in which healthy matched donor cells are transplanted into the patient and the enzyme is then secreted continuously from the donor cells; and chaperone therapy, which encompasses the use of competitive inhibitors at sub-inhibitory concentrations to stabilize the mutant enzyme, thereby extending the half-life and improving catalysis. Even though treatments are available for 11 LSDs, most of these disorders are managed symptomatically and patients only receive supportive care due to the inability to treat neurological symptoms [64].

Most importantly, even when therapies are available, especially ERTs, they are only successful in the somatic tissues of the body and cannot cross the blood–brain barrier (BBB); therefore, they fail to treat neurological deficits, which are among the most debilitating symptoms of many LSDs. Once neurological damage has occurred, it is extremely difficult to revert the phenotype. Thus, obtaining the correct enzyme dose in the brain is a major therapeutic goal. About two thirds of LSDs have neurological involvement [59]. This is why small-molecule drugs are being developed to cross the BBB, even though, so far, none reliably reach the brain. However, gene therapies that directly target the CNS are promising.

### 3.2. Mucopolysaccharidoses

One of the subgroups in which neurological symptoms are the most prevalent is the mucopolysaccharidoses (MPSs), which represent approximately 30% of LSD cases [69]. Seven major MPSs are currently known (MPSI, II, III, IV, VI, VII and IX), which result from mutations in the genes that code for one of 11 acid hydrolases involved in the degradation of GAGs. Each individual enzyme deficiency underlies one particular MPS (for instance, four different deficiencies trigger an equivalent number of MPS III disorders) [70] (Table 2). As these lysosomal enzymes fail to fulfill their function, the compounds accumulate in cells and tissues, which then causes progressive damage and a variety of clinical multi-organ manifestations, such as cardiovascular disease, respiratory problems, skeletal abnormalities and premature death, but the spectrum and severity of the disease manifestations vary between and within the MPS types [8,71]. These compounds can also accumulate outside of the lysosomes, thereby activating inflammatory pathways and an innate immune response via the tool-like receptor 4 and the complement system. Aspects such as neuroinflammation, short bones and aortic fragmentation can also arise due to this inflammatory response [8].

MPSs are heterogeneous and multisystemic diseases and manifestations vary not only between the subtypes but also within the same subtype. These characteristics affect the quality of life and lifespan of the patients. Clinically, MPS patients can be classified as having a “visceral phenotype”, a “neurodegenerative phenotype” or a “skeletal phenotype”, depending on the subtype of the disease. In general, MPS types I, II, VI and VII present with coarse facies, visceromegaly (hepatosplenomegaly), hernia, upper airway obstruction, joint stiffness, heart disease and other skeletal deformities as the main group characteristics. Due to these manifestations, these MPSs are usually classified as the group with “visceral phenotype”. A short stature is present in MPS I, II and VII patients. Furthermore, corneal clouding is also very frequent in all of these subtypes, except for type II, in which hearing loss is marked [8]. MPS III patients belong to the group with “neurodegenerative phenotype”, in which the clinical manifestations of the groups that were referred to above are mild but there is a marked neurodegeneration, which usually starts between 3 and 5 years of age and is accompanied by behavioral disturbances and hyperactivity. Finally, the “skeletal phenotype” is a characteristic of MPS IV patients, who show skeletal dysplasia and many other bone problems. They are mentally normal and have a short stature. MPS IX is not included in these three groups because the main clinical manifestation is the presence of joint swelling and synovial masses [8].

All subtypes are monogenic diseases that are transmitted in an autosomal recessive way except for MPS II, which is X-linked. In general, nonsense and frameshift mutations seem to lead to a more severe disease, while missense mutations are associated with more attenuated forms. Splicing mutations are generally associated with severe disease forms, but when the normal transcript is produced (even in small amounts), a milder phenotype can be present. This genotype–phenotype correlation can help to predict phenotype, which is very important for MPS I patients, for example, to ensure that the correct treatment option is applied. However, it is difficult to predict that the phenotype on an individual basis. This is why it is important to study the impact of each mutation at the cDNA and protein level, as well as develop new biomarkers for the assessment and follow-up of treated and untreated patients [72,73].

MPS type I is the most frequent form of MPS and results from mutations in the *IDUA* gene that codes for α-L-iduronidase (EC 3.2.1.76). A deficiency of this enzyme results in the lack of the degradation of dermatan and heparan sulphates (DS/ HS), which leads to their progressive accumulation. A wide range of phenotypic involvement exists, including three major recognized clinical entities: Hurler (MPS IH; OMIM #607014), which is the most severe; Scheie (MPS IS; OMIM #607016), which is milder; and Hurler–Scheie (MPS IH/S; OMIM #607015), which has an intermediate phenotype [74]. The incidence of MPS I is estimated to be approximately 1:100,000 births (reviewed in [73]). To date, at least 320 mutations in *IDUA* are known, of which 15.3% are splicing mutations ([75]; Table 2). The early initiation of treatment, as for all treatable LSDs, results in more favorable outcomes. For this subtype, treatment options include HSCT, which is the gold standard for severe forms of the disease and for young children in the early stages of Hurler syndrome, and ERT with recombinant laronidase (Aldurazyme^®^, Genzyme), either alone or in combination [73,76,77,78]. However, the diagnosis of MPS I is often difficult, particularly for patients with attenuated phenotypes, which results in the delayed introduction of treatment. Gene therapy for MPS I is still only in the preclinical stages of development [77].

MPS II, also known as Hunter syndrome (OMIM #309900), is caused by mutations in *IDS* gene, which result in a deficiency of iduronate-2-sulfatase activity (EC 3.1.6.13). This decreased activity leads to intracellular and extracellular accumulation of HS and DS in various organ systems, as in MPS I. This disease is the only MPS that is not inherited in an autosomal recessive manner but rather has an X-linked inheritance [73,79]. So far, at least 739 mutations in *IDS* are known, of which 8.8% are splicing mutations ([75]; Table 2). These genetic variations result in different phenotypes of the disease, which can be classified as severe or attenuated. The severe form affects about 60% of patients and has CNS involvement. The overall estimated incidence of MPS II is 1:162,000 live male births [79].

The two approved treatments for MPS II are ERT with recombinant human IDS infusions of idursulfase (Elaprase^®^, Shire) and HSCT, which has been shown to have neurological benefits in MPS II patients.

Sanfilippo syndrome, or MPS III, can be differentiated from the other types due to the predominance of CNS disease [59,80]. The main compound that is accumulated is HS. Depending on the mutated gene and, consequently, the associated enzyme deficiency, this type can be classified as: MPS IIIA (OMIM #252900), with mutations in the *SGSH* gene; IIIB (OMIM #252920), when the mutations are in *NAGLU*; IIIC (OMIM #252930), which is caused by mutations in the *HGSNAT* gene; or IIID (OMIM #252940), with mutations in *GNS*. To date, numerous mutations have been identified in each of the four genes, 2.5%, 3.1%, 17.6% and 14.3% of which affect the splicing process for subtypes A, B, C and D, respectively ([75]; Table 2). Somatic symptoms are mild, even though hepatosplenomegaly is often present but not usually diagnosed clinically, and cardiac problems are rare (reviewed in [81]). As HS accumulates primarily in the brain, classical ERT, which is the most successful strategy for other non-neurological LSDs, may not be effective. The BBB limits the availability of the enzyme in the brain and IT and intracerebroventricular (ICV) administrations are very invasive strategies that have a number of associated problems. Clinical trials have been conducted to investigate various methods for ERT delivery to the CNS; however, they have been shown not to promote neurocognitive benefits [82,83,84]. A recent clinical trial of MPS IIIA patients using IT administration for the defective enzyme showed a reduction in HS and GAG levels in the treated patients. Still, the primary neurocognitive endpoint was not met [83]. Currently, there are no available treatments for this syndrome. Most efforts are palliative and focus on regulating behavior (aggressiveness, hyperactivity, etc.) and sleep disturbances. However, a number of therapies are now being developed and evaluated, such as HSCT, gene therapy, SRT and anti-inflammatory therapies (reviewed in [80,85]).

MPS IV, also known as Morquio syndrome, is caused by the impaired degradation of keratan sulphate (KS). Two enzyme deficiencies are known to lead to this syndrome: N-acetylgalactosamine-6-sulphatase (GALNS; EC 3.1.6.4), which causes Morquio syndrome type A (OMIM #253000), and β-galactosidase (EC 3.2.1.23), which causes Morquio syndrome type B (OMIM #253010). To date, at least 378 mutations are known for MPS IVA, of which 10.3% are splicing mutations, and 265 are known for MPS IVB, 8.3% of which are known to affect the splicing process ([75]; Table 2). Both forms of MPS IV have skeletal dysplasia, very short stature, ligamentous laxity/joint hypermobility and odontoid hypoplasia as major characteristics. Most patients are mentally normal [70,86]. Nevertheless, neurological involvement can also occur in severe cases and can be life-threatening, with the affected individuals not normally surviving past the second or third decade of life. Those patients with milder forms of the disorder usually survive to adulthood, even though their life expectancy may be reduced [8]. ERT using recombinant human GALNS, elosulfase alfa (Vimizim^®^; BioMarin Pharmaceutical Inc) and HSCT are the treatment options for MPS IVA (reviewed in [87]). There are no therapies currently available for MPS IVB.

MPS type VI, or Maroteaux–Lamy syndrome (OMIM #253200), results from a deficiency of arylsulfatase B (N-acetylgalactosamine-4-sulfatase; EC 3.1.6.12), which is caused by mutations in the *ARSB* gene. This deficit results in the pathological accumulation of DS in most organs and systems. The incidence estimates range from 1:77,000 to 1:278,000 live births. Presently, 229 mutations in *ARSB* are known, of which 5.7% affect the normal splicing process ([75]; Table 2). As with MPS IV, a purely somatic disease occurs with no cognitive involvement. Patients present within a spectrum of clinical severity: when they have a severe case of the disease, i.e., showing the onset of symptoms before 2 or 3 years of age and impaired mobility by 10 years of age, usually die in second or third decade of life; when the disease is attenuated, patients have a later onset of symptoms and tend to be diagnosed either in their teens or in early adulthood [88]. ERT with galsulfase (Naglazyme^®^, BioMarin Pharmaceutical Inc) is currently the recommended first-line treatment for MPS VI, although there have been various studies published on the positive effects of HSCT and the combination of the two treatments on MPS VI patients (reviewed in [89]).

MPS VII, also known as Sly syndrome (OMIM #253220), is a rare type of MPS that is characterized by the lack of the β-D-glucuronidase enzyme (EC 3.2.1.31) due to mutations in the *GUSB* gene. This deficiency causes an accumulation of DS, HS and chondroitin sulphate (CS) proteoglycans, which are mainly sulfated in the 4 (C4S) and 6 (C6S) positions, in multiple tissues. MPS VII patients are phenotypically heterogeneous but there are a few common features that can be recognized, including short stature, coarse facial features, corneal clouding, hydrocephalus, skeletal deformation and cardiac diseases, similar to those features that are observed in MPS I and II. Interestingly, a distinguishing feature is observed in this subtype: hydrops fetalis, which is an abnormal accumulation of bodily fluids in several tissues [90,91]. To date, 81 mutations have been identified in *GUSB*, 7.4% of which are splicing mutations ([75]; Table 2). For the non-neurological manifestations of MPS VII, ERT with vestronidase alfa (Mepsevii™, Ultragenyx, Novato, CA, USA), which was approved by the FDA in 2017, is the recommended therapeutic approach [92]. As for the other types of MPS, HSCT has also been studied in MPS VII patients but no definitive conclusions about its therapeutic efficacy have yet been drawn due to the limited data (reviewed in [93]).

Finally, MPS IX, also known as hyaluronidase (EC 3.2.1.35) deficiency (OMIM # 601492), is caused by mutations in the *HYAL1* gene, which results in the accumulation of hyaluronan. It is an ultra-rare type of MPS and, to date, only four patients have been reported worldwide: one patient in the original report was diagnosed in 1996 and the three other patients belonged to a second family, who were diagnosed in 2011 [94,95]. All reported patients with MPS IX presented with joint and skeletal problems. According to the data that were collected from these patients, there are only seven mutations that are known to be responsible for this disease, none of which affect the splicing process ([75], Table 2).

Altogether, excluding the ultra-rare MPS IX, which has no associated splicing defects, 3% to 18% of the currently described mutations are known to disrupt the normal pre-mRNA splicing, depending on the MPS type being considered. This reinforces the need for a deeper study on the effects of this type of mutation, but it also makes them great candidates for splice modulation approaches. While 11 different MPSs exist and only 5 of them have approved therapeutic approaches, the need for additional treatment options is real. It is also worth mentioning that, even for the diseases that do have treatments available, the currently approved drugs fail to address CNS lesions, thus allowing for the neuropathological progression of the disorder and the resultant neuropsychiatric manifestations [96]. In fact, the development and delivery of effective treatments for these neurological and psychiatric signs and symptoms are universal hurdles that are faced not only by MPSs, but also by virtually every other LSD. This is why so many different therapeutic approaches are either being developed or are under evaluation for this group of disorders, from substrate reduction to gene therapy [97]. Also included among those possibilities are patient-tailored, mutation-specific approaches, which take advantage of the current knowledge on the molecular basis of these disorders to design a drug which holds potential to surpass the molecular defect that underlies pathology in one particular patient. Ultimately, there is even room for the so-called N-of-1 therapeutics, in which a drug is specifically designed for the treatment of just one patient.

## 4. RNA-Based Therapeutic Approaches for MPS Mutations

Altogether, there are at least 226 MPS-causing mutations that affect the pre-mRNA splicing process [75]. These mutations can occur in *cis*-acting elements, including 5′ss and 3′ss, GU-AG canonical nucleotides, the Py tract, branch point sequence, ESE, ESS, ISE and ISS, which affects their interaction with *trans*-acting factors (SR family proteins and hnRNPs). These mutations can have a higher frequency worldwide, can be identified in a small number of families or they can be unique. While some notable exceptions have been recognized for a few LSDs [9], no MPS-related splicing mutations have yet been identified as being particularly prevalent among affected individuals and/or specific populations. Nevertheless, MPS-causing mutations are good candidates for splicing modulation approaches for several reasons, which we have already listed. Over the following sections, we summarize functional studies that have focused on MPS-causing mutations that affect splicing, as well as the studies that we are aware of that have attempted the correction and/or amelioration of MPS disease phenotypes through splice modulation.

### 4.1. Functional Studies of Splicing Mutations and Development of Therapeutic Approaches Using Antisense Oligonucleotides: The MPS II Example

So far, 739 MPS II causal mutations have been reported in the *IDS* gene (OMIM *309900), 65 of which have been described as affecting splicing (around 8.8%) [75]. In a study that was published in 2015, Matos et al. performed an extensive functional analysis on three *IDS* gene splicing mutations in order to better understand how and why splicing is altered and they subsequently addressed the *in vitro* correction of one of them using splicing-related ASOs [98,99]. Two of them, c.257 C>T and c.241 C>T, are located in exon 3 and activate a cryptic splice site in this exon. The third, c.1122 C>T, is located in exon 8 of *IDS* and is responsible for the creation of a new 5′ss, which leads to a shorter transcript than wild-type.

This is particularly relevant since only two of these disease-causing variants had previously been characterized at cDNA level and shown to disrupt the normal *IDS* splicing process: c.257 C>T and c.1122 C>T. The third, while previously reported, had only been analyzed at the gDNA level and incorrectly classified as a nonsense mutation [100]. Reporter minigenes were used as tools to perform these functional analyses. In fact, there is a significant number of papers on the efficacy of *in silico* predictors, which directly compare the bioinformatic results to those that were obtained with reporter minigenes, taking the latter as “controls”, and only analyze patient RNA when available [101]. This is why the effects of intronic or exonic mutations on splicing should ideally be assessed both by *in silico* tools and through the construction and transient expression of minigenes that harbor the variants under analysis.

Moreover, the splicing regulation of exon 3 has also been addressed using mutant minigene analysis and overexpression/silencing assays. It was observed that SRSF2 and hnRNP E1 could be involved in the use and repression of the constitutive 3′ss of exon 3, respectively [98]. These two regulatory elements, SRSF2 and hnRNP E1, were overexpressed or silenced in the Hep3B cell line that was transfected with either wild-type (WT) or mutant minigenes. It was verified that the choice of the constitutive 3′ss of *IDS* may be dependent on an ESE site that is recognized by SRSF2, which is compromised by the presence of the mutation in this region and also affects the binding of the splicing silencers hnRNP E1 and E2. The correction of both mutations was not attempted because, in both cases, the full-length transcript leads to the production of aberrant proteins that arise from a missense (c.257 C>T) or a nonsense (c.241 C>T) mutation [98]. However, the studies that were performed may still be of use to the design of ASO therapeutic strategies that involve this exon.

**Figure 5:**
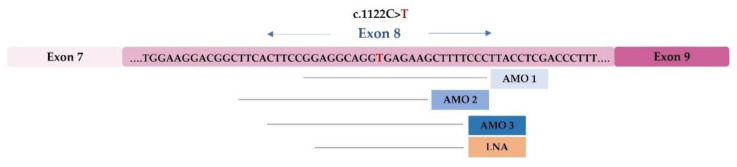
Antisense oligonucleotide (ASO) treatment for a MPS II-causing mutation: schematic representation of the *IDS* exon 8, in which the c.1122C>T nucleotide change is located (marked in red). The underlined sequences represent each blocking AMO or LNA that was designed for the different regions of the exon. Abbreviations: AMO, antisense morpholino; LNA, locked nucleic acid.

For the c.1122 C>T mutation, which has a silent effect on the amino acidic sequence, the possibility of redirecting the transcript processing using modified ASOs was tested in patients’ fibroblasts (Figure 5). Four ASOs were used, three 2′-O-methyl (2′OMe) and one locked nucleic acid (LNA), all of which were complementary to the region of the newly created 5′ss in order to block the access of the splicing machinery to the mutant mRNA, thus preventing the formation of the mutant transcript. Quite unexpectedly, however, this treatment failed to abolish the abnormal transcript and instead resulted in the appearance of another aberrant splicing product that corresponded to the total skipping of exon 8. Furthermore, the transfection of these ASOs in control fibroblasts also led to the appearance of the aberrant transcript that was observed in the patients’ cells, which showed that oligonucleotides masked an important *cis*-acting element for the 5′ss regulation of exon 8 [98].

Overall, the importance of functional studies for understanding the pathogenic consequences of mis-splicing became evident from these results. Moreover, this study highlighted the difficulty in developing antisense therapies involving regions of genes that are under complex splicing regulation.

### 4.2. Development of Therapeutic Approaches Using Modified U1 snRNA Vectors: The MPS IIIC Example

In 2014, Matos et al. showed that a modified U1 snRNA could be a promising tool for the treatment of splicing mutations in MPS IIIC patients. This was actually the first published study that assessed the potential of modified U1 snRNAs to correct of splicing mutations, not only in MPSs but also in the larger LSD field [102].

**Figure 6:**
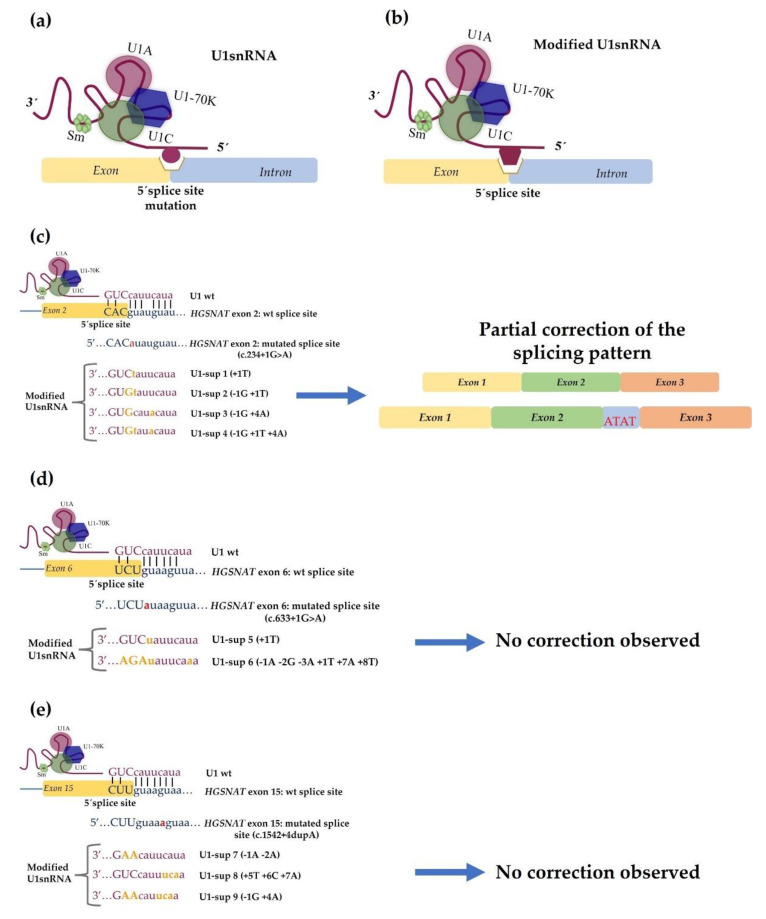
Therapeutic approach using modified U1 snRNA vectors: (**a**) the 5′ region of the U1 snRNA is involved in the recognition of the 5′ss. A mutation in this site compromises the binding of this molecule and the normal splicing process cannot occur; (**b**) a strategy for recovering the normal splicing process is the application of modified U1 snRNA to improve the recognition of the mutated 5′ss; (**c**–**e**) therapeutic approaches with different U1 snRNAs to correct the pathogenic effects of the splice site mutations in the *HGSNAT* gene (c.234+1G>A, c.633+1G>A and c.1542+4dupA). For the mutation described in (**c**), a partial recovery from the splicing defect was observed after treatment with the fully adapted U1 snRNA (U1-sup4). After sequence analysis, two different sequences were observed: one with a normal splicing pattern and another that included the first four base pairs of the intron 2 (ATAT). For the other two mutations at the 5′ss of the *HGSNAT* gene, no correction was observed after the application of the modified U1 snRNAs. Upper case letters show exonic nucleotides and lower case letters denote intronic nucleotides. Base pairing is indicated by vertical lines. The mutant nucleotide is highlighted in red and the changed nucleotides in the U1 sequence are illustrated in orange.

That study included five patients who carried four different mutations: c.234+1G>A, c.633+1G>A and c.1542+4dupA, which affect the donor splice site, and c.372-2A>G, which affects an acceptor splice site of the *HGSNAT* gene. For the first three mutations, different modified U1 snRNAs were designed to recognize the mutated site (Figure 6).

Again, the *in vitro* assessment was started by checking whether the splicing patterns that were observed in patients’ fibroblasts could be reproduced *in vitro* in an artificial system, which would allow for the subsequent functional analysis of each target mutation. In order to reproduce the splicing defects in a cellular model, several mutant minigenes were constructed and transfected in COS-7 cells. Post-transfection cDNA analysis and sequencing disclosed that the minigene-derived splicing patterns closely resembled the patterns that were observed in the control and patients’ cDNAs, which were obtained from the fibroblasts that had been previously analyzed. This observation further supported that those minigenes were reliable tools for testing and optimizing the overexpression of the modified U1 snRNAs to correct the splicing defects. So, several U1 constructs were generated with different degrees of complementarity to each mutated donor splice site. However, the splicing correction was not observed when they were tested in these artificial systems in all cases.

For the c.234+1G>A minigene, an expected band for the normal splicing was observed after co-expression with three of the five U1 snRNAs that were being tested; however, after sequence analysis, it was possible to observe that the fragment included exon 2 and the first four base pairs of intron 2 due to the use of an alternative downstream donor site (Figure 6c).

For the mutant c.633+1G>A minigene, an apparently normal band was detected with the overexpression of the U1 that matched all nucleotides of the mutated donor splice site. Yet again, the sequence analysis showed that, apart from exon 6, the first four nucleotides of the intron 6 were included. A band that corresponded to the skipping of exon 6 was also observed (Figure 6d).

In the c.1542+4dupA mutant minigene, when the co-transfection of the totally complementary U1 was performed, no correction was achieved, as the resulting fragment included not only exon 15 but also the first four nucleotides of intron 15. The inclusion of intronic nucleotides in all cases was due to the presence of a “gt” dinucleotide in positions +5 and +6 (Figure 6e).

Despite these results and taking into account that the minigenes only included partial intronic sequences that could lack some splicing regulatory sites and that they were assayed in non-human cells, modified U1 snRNAs were tested on patient-derived fibroblasts. For the c.234+1G>A mutation, a partial correction (almost 50%) was observed when the totally complementary U1 was transfected: one sequence demonstrated normal splicing and the other included the first four base pairs of intron 2 (as detected in the minigene approaches with COS-7 cells). However, no improvement in enzyme activity was observed. In the other patient fibroblasts (mutations c.633+1G>A and c.1542+4dupA), no effects of any modified U1 snRNAs were observed on the endogenous splicing process.

### 4.3. Identification and Characterization of Novel Splicing Defects and Assessment of Their Amenability for Splicing Correction Therapeutic Approaches: The MPS I Example

While there are only two publications on the design of innovative approaches for the correction of specific splicing defects in MPSs, to the best of our knowledge, many other MPS-causing mutations could also be amenable to splicing correction therapeutic approaches, as demonstrated by the significant number of splicing defects that have been (already) identified in this group of pathologies (Table 2). Moreover, as in DMD, other mutations besides the splicing mutations could be corrected with ASOs, namely the deletions and insertions that cause frameshift and for which exon skipping approaches could be applicable. Thus, many other studies could be designed to assess the feasibility of ameliorating the phenotypes of these multisystemic diseases by “simply” either correcting, skipping or partially recovering their underlying defects. The recent developments in the broader RNA therapeutics field, together with the growing number of splicing modulation therapeutics that have either been approved or are under development, will certainly contribute to increase the number of studies using this sort of approaches and extend the catalogue of genetic diseases to which they apply.

**Figure 7:**
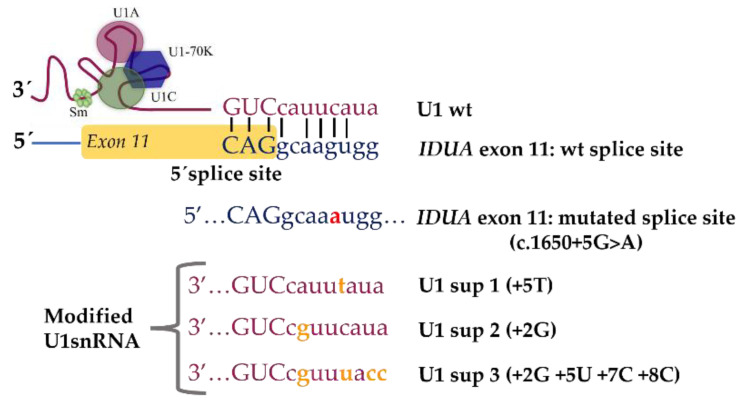
Therapeutic approach using modified U1 snRNA vectors. Different U1 snRNAs were designed to correct the pathogenic effects of the splice site mutation c.1650+5G>A in the *IDUA* gene. Upper case letters show exonic nucleotides and lower case letters denote intronic nucleotides. Base pairing is indicated by vertical lines. The mutant nucleotide is highlighted in red and the changed nucleotides in the U1 sequence are illustrated in orange.

In our lab, for example, we are also addressing another MPS-causing mutation, which is known to disrupt splicing: the c.1650+5G>A mutation in the *IDUA* gene (Figure 7). This single nucleotide change leads to exon 11 skipping and, when present in homozygosity or compound heterozygosity, causes MPS I. Being a 5′ss mutation, this pathogenic variant could be an excellent target for mutation-specific U1 snRNA-mediated therapeutic approaches. Thus, we performed this antisense snRNA therapeutic strategy on fibroblasts of a MPS I patient harboring the 5′ss mutation c.1650+5G>A in compound heterozygosity with a nonsense mutation (c.1205 G>A; p.W402X) in intron 11, which leads to the exon 11 skipping of the *IDUA* gene. Briefly, we constructed three different U1 variants with increased complementarity to the mutated 5′ss. Unfortunately, when they were transfected in the patients’ fibroblasts, no correction was achieved. Instead, it was still possible to observe the skipping of exon 11 (unpublished data).

The 5′ss is a very important sequence as it is a key factor in influencing not only the recognition of the donor splice site by U1, but also the overall success of the U1 therapeutic approach. This sequence can have a degenerative pattern feature and does not always conform to the consensus sequence (CAG/GURAG; R-purine) [103,104]. Therefore, not all positions of the sequence are equally important for enabling recognition by U1 and ensuring correct splicing. Various base pair combinations within the 5′ss can increase the U1 binding affinity [105].

Having this in mind, we are now performing further investigations. We started with one of the most obvious possibilities: the hypothesis that the absence of correction for the c.1650+5G>A mutation was caused by a low transfection efficacy. An interesting approach would be to test the therapeutic recovery of the mutation using a viral transduction technique. Viral vectors are considered significantly more efficient and less toxic than other delivery systems, namely cationic lipid transfection reagents such as Lipofectamine^®^. In fact, the viral transduction of U1 constructs in patients’ fibroblasts has already been successfully applied for some diseases, allowing for the total or partial recovery of mis-spliced transcripts [106,107,108]. This is what we are currently testing in fibroblasts from MPS I patients carrying this splicing mutation. Other alternatives include testing the effects of modified U6 snRNA vectors in a similar way to that tested for the U1 snRNA vectors. Indeed, U6 snRNA has also been described as essential for proper splicing since its interaction with nucleotides at positions +4 to +6 of the splice donor site is necessary for the correct recognition of the exons at the 5′ss [109,110]. There is a published example in which only the co-application of adapted U1 and U6 isoforms corrected the splice defects that were caused by a +5 mutation [105].

Whatever the MPS we chose, the possibilities are numerous and diverse, as the catalogue of splicing defects known to cause it is vast (Table 2). Nevertheless, most of those variants are not particularly frequent among affected families. In fact, many of them are unique or rare. This could be an obstacle not only to the development of this sort of approaches, but also ultimately to making sure that those approaches that succeed eventually reach the clinic.

## 5. Challenges for the Development of Splice Modulation Approaches for MPSs

Regardless of these hurdles, MPSs, as with virtually any other LSD, are excellent candidates for splicing modulation for a number of reasons. First, they are monogenic diseases whose molecular bases have been under the lens of several teams around the world for many decades and knowledge about them has increased tremendously during this time. Second, and perhaps most importantly, it is assumed that a threshold enzyme activity of approximately 10% is sufficient to prevent storage in LSDs [111,112]. This means that even a partial recovery could be sufficient to promote a clinically relevant effect.

Altogether, the possibilities are multiple and worth addressing. Still, there are at least two major issues that we need to address in order to ensure that this sort of therapeutic approach fulfils its full potential in the LSD field. The first, and most obvious, issue is the need for appropriate animal models in order to test these approaches *in vivo*.

### 5.1. Existence of Disease-Relevant Models

As important as cell models may be, a significant part of the efforts to demonstrate the therapeutic potential of any drug relies on studies with model organisms. The preclinical studies of adequate animal models are a major prerequisite, not only as proof of efficacy but also for safety and toxicity assessments, which are essential for the design of subsequent clinical trials. As previously discussed, the proper *in vivo* testing of splice modulation therapeutics requires the development of animal models that carry the specific splicing mutations. In fact, even though genetic models for MPSs encompass a wide range of biological systems [113,114,115] thanks to the numerous advances in mutagenesis techniques that have markedly improved the efficiency of model generation, knockout or transgenic mouse models that carry null mutations remain the gold standard within the field. It is important to notice, however, that while efforts should be made to develop suitable animal models, this may not be a straightforward task given the differences in the sequences that are involved in the overall splicing processes in different species [9]. Furthermore, the numerous species-specific differences that exist in orthologue-coding sequences may also hamper the process of animal model generation.

### 5.2. Design and Development of Effective Delivery Strategies

While the most obvious difficulty in terms of delivery is probably the BBB, which prevents patients with MPSs that involve the CNS from benefiting from several of the possible therapeutic approaches, including those which are already on the market, brain delivery may actually be feasible for some specific splicing modulation approaches. In fact, taking into account the latest findings on the wide distribution of ASOs after IT administration and its safety and tolerability, splicing modulation approaches that rely on ASOs hold a great promise for clinical translation. Nevertheless, the delivery of modified U1 snRNAs to the brain remains a pending issue. It is also important to note that brain delivery is far from being the only concern when it comes to promoting the clinical translation of this sort of approaches. There are other target tissues/organs that need to be taken into account when considering MPS-tailored approaches, namely the skeletal system. In fact, skeletal pathology is a huge burden in many MPSs and the currently available therapies fail to prevent or resolve it. The same is true for cardiovascular targeting, even though cardiovascular disease is not as prevalent in MPSs as skeletal pathology. Thus, both bone- and heart-targeting of therapeutic molecules are issues to be considered when designing splicing modulation approaches for MPS. Again, one possibility is to take advantage of the cell-specific receptors that can be targeted for uptake into these particularly impervious tissues [61].

### 5.3. Accurate Characterization of Disease-Causing Variants at mRNA Level

Finally, there is yet another issue that should not be forgotten: our efforts to correct specific pathogenic variants should also be accompanied by a serious attempt to characterize each novel disease-causing variant more accurately. While this may sound strange in a post-genomic era in which NGS allows for multiple genes to be sequenced in parallel, assuring a faster and more efficient identification of pathogenic variants while saving time and resources, the need for in-depth molecular characterization remains an issue [116]. In fact, even though NGS technologies have contributed to greatly to enlarging the catalogue of known disease-causing variants and have actually broadened the overall number of known genetic diseases (for example, the recently identified MPS type X was actually identified through exome sequencing,), many of those variants need to be further investigated. This is particularly relevant for the mutations that affect splicing, which have to be functionally characterized and their impact evaluated at the molecular level. In fact, DNA variants that affect mRNA expression and processing are often missed or poorly characterized, not only because they are only analyzed at the genomic level but also because certain mRNA species tend to be subjected to degradation. A recent example in the field came from our own experience in the molecular characterization of LSD patients. For example, we recently demonstrated that an *NPC1* silent variant, which was previously classified as a non-pathological polymorphism (p.V562V), actually induces exon 11 skipping, which then leads to the appearance of a premature stop codon and underlies juvenile Niemann–Pick type C disease. This work relied on a series of molecular studies and led us to revisit other Portuguese patients who had been molecularly screened for the *NPC1* gene but for whom it was not possible to establish a definitive diagnosis. By doing so, we found a second patient with a clinical presentation of Niemann–Pick type C who harbored the silent p.V562V in heterozygosity with another known disease-causing mutation [117], thus highlighting the interest of reanalyzing existing test results in known disease genes **[116]**.

Plus, a better understanding of the fine mechanisms that regulate AS will also allow for a more effective targeting of those processes, thus contributing to the design and development of novel and more effective tools for therapeutic splicing modulation.

## 6. Concluding Remarks

Several lines of evidence support the *in vivo* effectiveness of RNA-based therapies in recovering aberrant splicing and, while exploratory, the studies on MPSs tend to follow this trend. Overall, the results that were reviewed in this paper further encourage the preclinical development and testing of this sort of approaches for this group of diseases, which so far either completely lack effective therapeutic options or have an urgent need for less expensive and more effective treatment. Still, in order for these approaches to reach the clinic and fulfill their therapeutic potential, several measures need to be undertaken both before and after the *in vitro* assessments. In fact, in an era in which a single genetic analysis allows us to sequence a huge number of genes and provide fast and reliable diagnoses, DNA variants that affect mRNA expression and processing are often still missed or their effects are poorly characterized. Thus, any efforts to address the therapeutic potential of splice modulation approaches should probably start earlier, with the proper molecular analysis of disease-causing pathogenic variants, in order to better characterize the incidence of splicing mutations and better understand their impacts at the molecular level. It is also mandatory to address the subsequent need for suitable animal models and better delivery systems for *in vivo* testing.

In addition, while not discussed in this review, there is another possible way to apply splicing modulation ASOs as a potential therapeutic approach for the treatment of MPSs: to deliberately skip or promote the skipping of disease-bearing exons. This is an approach that is somehow similar to that used for the treatment of DMD patients, which we briefly summarized in our introduction section (Figure 3). This would obviously require extra caution because the removal of whole exons or series of exons may be quite deleterious. Nevertheless, it could be feasible and even advantageous in some particular cases, as long as some key requirements are met. First, it would have to be checked whether the exon skipping under consideration would give rise to an in-frame protein product because it is mandatory to keep the remaining amino acid sequence intact. Then, it would also be necessary to check which protein domains would be affected by the change and how essential they are for protein function. Skipping an exon that codes for amino acids that are directly involved in the catalytic activity core of the enzyme, for example, may have a direct impact on protein function. Therefore, a careful bioinformatic analysis should be performed before considering this approach *in vitro*. Once attempted either in patient or model cell lines, a cautious analysis of the enzyme activity, location and expression should also be undertaken. While risky, this may be yet another route to targeting MPS diseases using splicing modulation approaches. Nevertheless, to the best of our knowledge, no-one has ever attempted this sort of therapeutic approach for MPS diseases.

## Figures and Tables

**Table 1:** Antisense oligonucleotides (ASOs) that are approved for Duchenne muscular dystrophy (DMD) and spinal muscular atrophy (SMA) treatment.

Brand Name	Drug	Year of Approval	Target Molecule	Treatment Result	Target Disease
Spinraza^®^, Biogen	Nusinersen	2016	*SMN2* mRNA	Induces the inclusion of exon 7 in the *SMN2* mRNA	Spinal muscular atrophy
Exondys 51™, Sarepta Therapeutics	Eteplirsen	2016	Dystrophin mRNA	Induces the exclusion of exon 51 of dystrophin mRNA	Duchenne muscular dystrophy
Vyondys 53™, Sarepta Therapeutics	Golodirsen	2019	Dystrophin mRNA	Induces the exclusion of exon 53 of dystrophin mRNA	Duchenne muscular dystrophy
Viltepso^®^, NS Pharma	Viltolarsen	2020	Dystrophin mRNA	Induces the exclusion of exon 53 of dystrophin mRNA	Duchenne muscular dystrophy
Amondys 45™, Sarepta Therapeutics	Casimersen	2021	Dystrophin mRNA	Induces the exclusion of exon 45 of dystrophin mRNA	Duchenne muscular dystrophy

**Table 2:** Classification of mucopolysaccharidose (MPS) subtypes.

MPS Type	Common Name(s)	Associated Gene	Enzyme Deficiency	Number of Mutations	% of Splicing Mutations	Treatment Options Available
I	Hurler, Scheie and Hurler–Scheie syndromes	*IDUA*	Alpha-L-iduronidase	320	15.3	ERT, HSCT
II	Hunter syndrome	*IDS*	Iduronate-2-sulfatase	739	8.8	ERT, HSCT
IIIA	Sanfilippo syndrome type A	*SGSH*	Heparan-*N*-sulfatase	163	2.5	-
IIIB	Sanfilippo syndrome type B	*NAGLU*	*N*-acetylglucosaminidase	256	3.1	-
IIIC	Sanfilippo syndrome type C	*HGSNAT*	Acetyl CoA glucosamine *N*-acetyltransferase	91	17.6	-
IIID	Sanfilippo syndrome type D	*GNS*	*N*-acetyl-glucosamine-6-sulfatase	28	14.3	-
IVA	Morquio syndrome type A	*GALNS*	*N*-acetylgalactosamine-6-sulfate sulfatase	378	10.3	ERT, HSCT
IVB	Morquio syndrome type B	*GLB1*	β -galactosidase	265	8.3	-
VI	Maroteaux–Lamy syndrome	*ARSB*	Arylsulfatase B	229	5.7	ERT
VII	Sly syndrome	*GUSB*	β-glucuronidase	81	7.4	ERT
IX	Hyaluronidasedeficiency	*HYAL1*	Hyaluronidase	7	0	-

## Data Availability

Not applicable.
